# Attenuation of muscle atrophy by an N-terminal peptide of the receptor for proteolysis-inducing factor (PIF)

**DOI:** 10.1038/bjc.2011.216

**Published:** 2011-06-14

**Authors:** K A Mirza, S M Wyke, M J Tisdale

**Affiliations:** 1 Nutritional Biomedicine, School of Life and Health Sciences, Aston University, Birmingham B4 7ET, UK

**Keywords:** proteolysis-inducing factor (PIF), PIF receptor, muscle protein degradation, cancer cachexia

## Abstract

**Background:**

Atrophy of skeletal muscle in cancer cachexia has been attributed to a tumour-produced highly glycosylated peptide called proteolysis-inducing factor (PIF). The action of PIF is mediated through a high-affinity membrane receptor in muscle. This study investigates the ability of peptides derived from the 20 N-terminal amino acids of the receptor to neutralise PIF action both *in vitro* and *in vivo*.

**Methods:**

Proteolysis-inducing factor was purified from the MAC16 tumour using an initial pronase digestion, followed by binding on DEAE cellulose, and the pronase was inactivated by heating to 80°C, before purification of the PIF using affinity chromatography. *In vitro* studies were carried out using C_2_C_12_ murine myotubes, while *in vivo* studies employed mice bearing the cachexia-inducing MAC16 tumour.

**Results:**

The process resulted in almost a 23 000-fold purification of PIF, but with a recovery of only 0.004%. Both the D- and L-forms of the 20mer peptide attenuated PIF-induced protein degradation *in vitro* through the ubiquitin-proteosome proteolytic pathway and increased expression of myosin. *In vivo* studies showed that neither the D- nor the L-peptides significantly attenuated weight loss, although the D-peptide did show a tendency to increase lean body mass.

**Conclusion:**

These results suggest that the peptides may be too hydrophilic to be used as therapeutic agents, but confirm the importance of the receptor in the action of the PIF on muscle protein degradation.

Half of all cancer patients experience a progressive loss of body mass due to depletion of adipose tissue and skeletal muscle. This process, called cachexia, is estimated to be responsible for at least 20% of cancer deaths through cardiac/respiratory failure (Inagaki *et al*, 1974) and the patients quality of life is also reduced through immobility. Tissue wasting is progressive and extensive, with loss of 85% of adipose tissue and 75% of skeletal muscle mass, when the weight loss reaches 30%, which is close to death (Fearon, 1992).

To provide appropriate therapy, it is necessary to understand the mechanism of tissue atrophy in cachexia. A number of factors, including cytokines such as tumour necrosis factor-*α* (Oliff *et al*, 1987) and interleukin-6 (Strassman *et al*, 1992), have been implicated as playing a role in the development of cachexia. In addition, our own studies have shown that those tumours that initiate muscle atrophy produce a novel sulphated glycoprotein of Mr 24 000 called proteolysis-inducing factor (PIF; Todorov *et al*, 1996a), which induces muscle wasting, both by depressing protein synthesis and by increasing protein degradation (Lorite *et al*, 1997). Protein degradation induced by PIF is mediated through an upregulation of the expression and activity of the ubiquitin-proteasome proteolytic pathway, which is considered to have the major role in the degradation of skeletal muscle in cachectic cancer patients (Lorite *et al*, 1998). The central mediator for the depression of protein synthesis and induction of protein degradation induced by PIF is phosphorylation of the RNA-dependent protein kinase (PKR) (Eley and Tisdale, 2007). Autophosphorylation of PKR causes activation, resulting in phosphorylation of its substrate eukaryotic initiation factor-2 on the *α*-subunit, leading to inhibition of translation initiation by preventing binding of the initiator methionyl-tRNA to the 40S ribosomal subunit. Activation of PKR also initiates protein degradation through the ubiquitin-proteasome pathway by increasing nuclear binding of the transcription factor nuclear factor-*κ*B (Eley and Tisdale, 2007). The action of PIF is mediated through an Mr 40 000 membrane receptor with high affinity (Kd 2.1–21 nmol l^−1^) present on muscle and liver, but not on kidney or adipose tissue (Todorov *et al*, 2007). Antisera reactive to a 19mer corresponding to the N-terminal amino acids of the PIF receptor was effective in attenuating weight loss in mice bearing the cachexia-inducing MAC16 tumour, through an increase in lean body mass, attributed to an increase in protein synthesis and decrease in degradation in skeletal muscle.

This study investigates the possibility of using the PIF receptor N-terminal 20mer to neutralise PIF action *in vitro*, as well as its ability to neutralise muscle atrophy in mice bearing the MAC16 tumour. Both the D- and L-versions of the peptide were investigated, as the D-form would be resistant to proteolytic cleavage and more suitable for *in vivo* studies. This study also reports a new method for the preparation of PIF from the MAC16 tumour, because of the reported difficulties in the isolation and identification of PIF (Wieland *et al*, 2007).

## Materials and methods

### Materials

Fetal calf serum (FCS), horse serum (HS), and Dulbecco's modified Eagle's medium (DMEM) were purchased from Invitrogen (Paisley, UK). L-[2,6-^3^H] phenylalanine (sp.act.37 Bqmmol^−1^) was from American Radiolabelled Chemicals (Cardiff, UK). Mouse monoclonal antibodies to 20S proteasome *α*-subunits and p42 were from Affiniti Research Products (Exeter, UK) and mouse monoclonal antibody to myosin heavy-chain type II was from Novocastra (via Leica Biosystems, Newcastle, UK). Polyclonal rabbit antibody to mouse *β*-actin was purchased from Sigma-Aldrich (Dorset, UK). Peroxidase-conjugated sheep antirabbit and antimouse antibodies were purchased from GE Healthcare (Bucks, UK), as were Hybond A nitrocellulose membranes. Enhanced chemiluminescence (ECL) development kits were from Pierce through Thermo Fischer Scientific (Northumberland, UK). The D- and L-amino-acid versions of the N-terminal 20 amino acids of the PIF receptor Asp-Ile-Asn-Gly-Gly-Gly-Ala-Thr-Leu-Pro-Gln-Lys-Leu-Tyr-Leu-Ile-Pro-Asn-Val-Leu were synthesised by Severn Biotech Ltd (Worcs, UK). For the D-amino-acid version, the amino acids were in the reverse order, so that the Leu became the N-terminal amino acid and the Asp the C-terminal.

### Animals

Fragments (1 × 2 mm) of the MAC16 tumour, obtained from donor animals with maximum weight loss, were implanted s.c. into the flank of pure strain male NMRI mice, obtained from our own inbred colony, by means of a trochar, as previously described (Bibby *et al*, 1987), Animals were fed a rat and mouse breeding diet (Special Diet Services, Witham, UK) and water *ad libitum*, and weight loss was evident 12–15 days after transplantation when the tumour became palpable. When the mice had lost ∼5% of their starting body weight they were randomised into groups of six to receive either D- or L-peptide or solvent (PBS) administered daily by i.v. injection. Both tumour volume and body weight were monitored daily and animals were terminated by cervical dislocation when the body weight loss reached 20%. All animal experiments followed a strict protocol approved by the British Home Office, and the ethical guidelines that were followed met the standards required by the UKCCR guidelines (Workman *et al*, 1998). After termination by cervical dislocation, the body composition was determined as previously described in detail (Russell *et al*, 2004). Carcasses were heated at 80–90°C for 48 h, or until a constant weight was achieved and the water content was determined as the difference between the wet and dry weights. Lipids were extracted with chloroform/methanol (1 : 1), ethanol/acetone (1 : 1) and diethyl ether and after evaporation of the solvent the fat content was determined from the weighed residue. The non-fat carcass mass was calculated as the difference between the initial weight of the carcass and the weight of water and fat.

### Cell culture

C_2_C_12_ murine myoblasts were maintained *in vitro* in DMEM supplemented with 10% FCS and glutamine under an atmosphere of 10% CO_2_ in air at 37°C. When the myoblasts reached about 80% confluency they were differentiated into myotubes in DMEM containing 2% HS, with medium changes every 2 days. Differentiation was complete in 3–5 days, and the myotubes remained viable for a further 4–5 days.

### Measurement of total protein degradation

This was performed as previously described (Whitehouse and Tisdale, 2003). Briefly, myotubes were labelled for 24 h with L-[2,6-^3^H] phenylalanine and then washed extensively in PBS followed by a further incubation for 2 h to eliminate degradation of short-lived proteins. Protein degradation was determined over a 24-h period in the presence of 2 mM non-labelled phenylalanine to prevent reincorporation of radioactivity, as a fraction of the total radioactivity incorporated into the myotubes.

### Measurement of proteasome activity

The functional activity of the 20S proteasome was determined as the ‘chymotrypsin-like’ enzyme activity, as previously described in detail (Whitehouse and Tisdale, 2003). Activity was determined by the release of aminomethyl coumarin (AMC) from the fluorogenic peptide succinyl-LLVY-AMC in the absence and presence of the specific proteasome inhibitor lactacystin (10 *μ*M). Only lactacystin-suppressible activity was considered to be proteasome specific. The activity was normalised to the protein content, which was determined using a standard colourimetric protein assay (Sigma, Dorset, UK).

### Purification of PIF

Solid MAC16 tumours (5 g) were homogenised in 25 ml 10 mM Tris-HCl, pH 8.0, on ice, and incubated with pronase (1 mg ml^−1^) overnight at 37°C. The soup was centrifuged at 4000 **g** for 15 min to remove the pellet, and the supernatant was concentrated to a volume of 1 ml against PBS using an Amicon Ultra-15 centrifugal filter (Millipore, Cork, Rep of Ireland) with a molecular weight cutoff of 1000. DEAE cellulose (2 g), previously washed with 100 mM Tris-HCl, pH 8.8 for 30 min and 10 mM Tris-HCl, pH 8.8 for another 30 min, was resuspended in 20 ml 10 mM Tris-HCl, pH 8.8, and was incubated with the protein concentrate for 2 h at room temperature. The PIF bound to the DEAE cellulose, which was sedimented by centrifugation at 4000 **g** for 15 min, and the PIF was eluted with 0.3 M NaCl in 20 ml 10 mM Tris pH 8.8, by incubation for 30 min. The DEAE cellulose was removed by centrifugation at 4000 **g** for 15 min, and the supernatant was concentrated to 1 ml against PBS using an Amicon Ultra-15 centrifugal filter with a molecular weight cutoff of 10 000. Proteolysis assays suggested that the concentrate still contained pronase. To inactivate this, the sample was heated to 80°C for 15 min. Two methods were investigated for the isolation of PIF: (1) Wheat germ agglutin agarose, based on the observation (Todorov *et al*, 1996b) that this lectin will bind the oligosaccharide chains of PIF. The sample was loaded onto the column and washed with 10 mM Tris-HCl, pH 7.4 and eluted with 0.1 mM *N*-acetylglucosamine. (2) Affinity chromatography using anti-PIF monoclonal antibody coupled with protein A on an Affi-Gel column. The retained proteins were eluted with 100 mM glycine-HCl, pH 2.5, as described (Todorov *et al*, 1996b).

### Western blotting

Samples of cytosolic protein (5–15 *μ*g), formed by centrifugation of cellular homogenates from the ‘chymotrypsin-like’ enzyme activity at 18 000 **g** for 10 min at 4°C, or from steps in the purification of PIF, were resolved on 12% SDS–PAGE at 180 V for ∼1 h, followed by transference onto 0.45 *μ*m nitrocellulose membranes. These were blocked with 5% Marvel in Tris-buffered saline, pH 7.5, for 1–2 h at room temperature (20S, p42) or at 4°C overnight. Both primary and secondary antibodies were used at a dilution of 1 : 1000 except for myosin (1 : 40). Incubation was for 2 h at room temperature and development was by ECL. Blots were scanned by a densitometer to quantify differences.

### Statistical analysis

Data are presented as mean values±s.e. for at least three replicate experiments. Differences in mean values between groups were determined by one-way ANOVA followed by Tukey–Kramer multiple comparison test. *P*-values <0.05 were considered significant.

## Results

**Figure 1:**
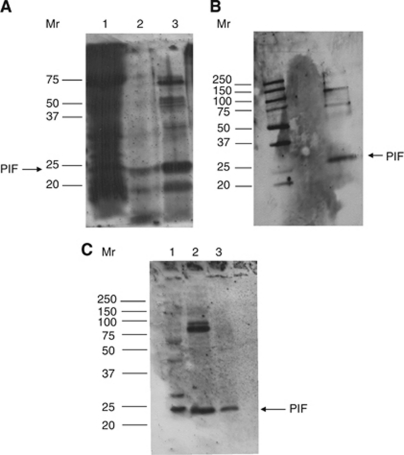
Purification of PIF from the MAC16 tumour (**A**) The tumour homogenate (lane 1) was incubated with pronase (1 mg ml^−1^) overnight at 37°C, and the PIF was concentrated by binding to DEAE cellulose (lane 2). Pronase was inactivated by heating to 80°C for 15 min (lane 3). The PIF was purified by affinity chromatography using either anti-PIF antibody (**B**) or WGA (**C**). In (**C**) lane 1 shows the tumour extract after heating to 80°C, lane 2 shows material not adherent to the WGA column while lane 3 shows retained material eluted with 10 mM Tris-HCl, pH 7.4 containing 0.1 M *N*-acetylglucosamine as for purification of the PIF receptor (Todorov *et al*, 2007). The figures are western blots using the anti-PIF monoclonal antibody (Todorov *et al*, 1996b)

A new purification scheme has been devised for PIF, the steps and yields of which are given in Table 1. Initially, the MAC16 tumour homogenate was subjected to pronase digestion, because PIF, unlike the majority of tumour proteins, is not affected by pronase, being predominantly carbohydrate in composition (Todorov *et al*, 1996a). Proteolysis-inducing factor can be recovered from the digest by binding to DEAE cellulose (Figure 1A), as the molecule has a high overall negative charge, but enzyme assays showed co-purification with the pronase, which would preclude further purification by affinity chromatography. To overcome this problem, the pronase was inactivated by heating at 80°C for 15 min, after which time western blotting revealed that the PIF was not destroyed by this treatment (Figure 1A). This allowed a final purification of PIF by affinity chromatography using an anti-PIF monoclonal antibody (Figure 1B) giving an overall purification of 22 936-fold (Table 1). Alternatively, the PIF can be purified by lectin affinity chromatography using wheat germ agglutinin agarose and elution with *N*-acetylglucosamine (Figure 1). However, the capacity of this type of affinity chromatography was less than with antibody and some of the PIF came through with the column washings (Figure 1).

**Figure 2:**
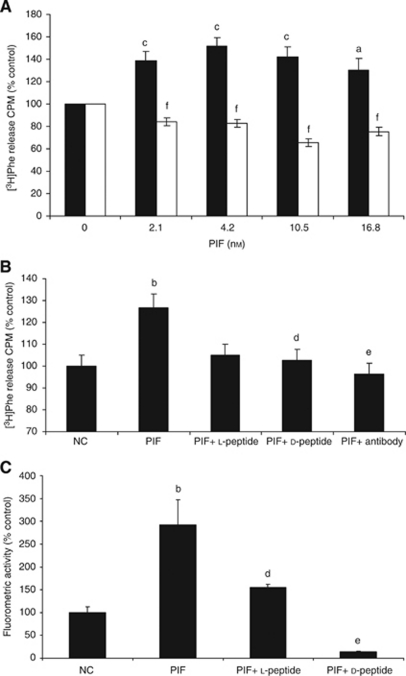
Effect of receptor peptides on protein degradation by PIF in C_2_C_12_ myotubes. (**A**) Total protein degradation after 24 h in response to PIF alone (▪) or in the presence of the L-receptor peptide (□) (10 *μ*M) added 2 h before the addition of PIF. (**B**) Comparison of D- and L-peptides (both 10 *μ*M) and anti-PIF antibody (10 *μ*g ml^−1^) on protein degradation induced by PIF (4.2 nM) over a 24-h period. (**C**) Effect of the D- and L-peptides (both 10 *μ*M) on the proteasome ‘chymotrypsin-like’ enzyme activity in response to PIF (4.2 nM) after 24 h incubation. The experiment was repeated three times. Differences from control are indicated as a, *P*<0.05; b, *P*<0.01; or c, *P*<0.001, while differences between groups are indicated as d, *P*<0.05; e, *P*<0.01; or f, *P*<0.001.

**Figure 3:**
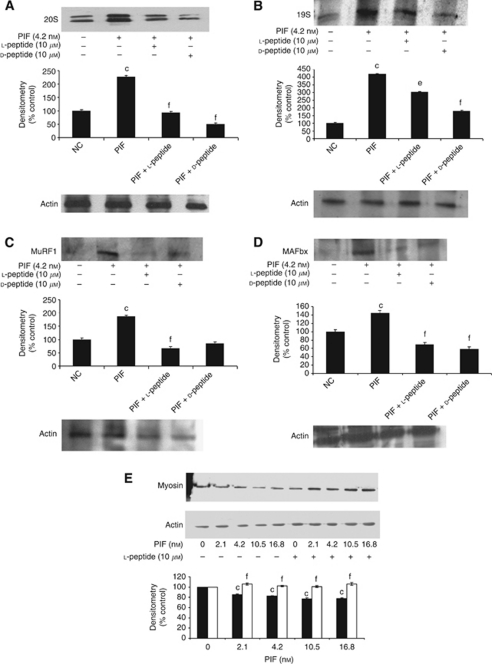
Effect of PIF-receptor peptides on expression of components of the ubiquitin-proteasome pathway in the presence of PIF (4.2 nM). Western blotting showing expression of the 20S proteasome *α*-subunits (**A**), p42, a component of the 19S regulator (**B**), the E3 ligases MuRF1 (**C**), MAFbx (**D**) in murine myotubes 24 h after addition of PIF in the absence or presence of the L- and D-peptides (both 10 *μ*M). The expression of the myofibrillar protein myosin with actin as loading control is shown in (**E**). The densitometric analysis is based on three separate blots, and values in the presence of PIF are indicated as (▪), while for myotubes co-incubated in the presence of the receptor peptide are shown as (□). Differences from controls are shown as c, *P*<0.001, while differences from PIF alone are shown as e, *P*<0.01 or f, *P*<0.001.

To determine the ability of the N-terminal 20mer of the PIF receptor to attenuate protein degradation induced by PIF, experiments were conducted in murine myotubes as a surrogate model of skeletal muscle. A dose–response curve for total protein degradation induced by PIF in murine myotubes in the absence and presence of the L-peptide is shown in Figure 2A. There was not a significant difference in protein degradation at concentrations of PIF between 2.1 and 10.5 nM, and the bell-shaped dose–response was less pronounced than in previous studies (Whitehouse and Tisdale, 2003). However, PIF was used for all further *in vitro* studies at a concentration of 4.2 nM to maintain continuity with other investigations. Protein degradation by PIF was completely attenuated by the L-peptide at all concentrations of PIF (Figure 2A). The D-peptide also attenuated total protein degradation in myotubes, as did the anti-PIF antibody (Figure 2B). To determine if the effect on total protein degradation was due to attenuation of the ubiquitin-proteasome pathway, functional activity was measured as the ‘chymotrypsin-like’ enzyme activity. As with total protein degradation PIF increased the ‘chymotrypsin-like enzyme activity (Figure 2C), and this was attenuated by both the D- and L-forms of the receptor N-terminal 20mer. Further support for the involvement of the ubiquitin-proteasome pathway was provided by the attenuation, by both the D- and L-forms of the receptor peptide, of the PIF-induced increase in the expression of the 20S proteasome *α*-subunits (Figure 3A), p42, an ATPase subunit of the 19S regulator (Figure 3B), the muscle atrophy promoting E3 ligases MuRF1 (Figure 3C), and MAFbx/atrogin-1 (Figure 3D). Myotubes treated with either the D- or L-forms of the peptide also showed an increase in expression of the myofibrillar protein myosin (Figure 3E). These results suggest that the PIF receptor N-terminal peptides may be effective in the attenuation of muscle wasting in cachexia.

**Figure 4:**
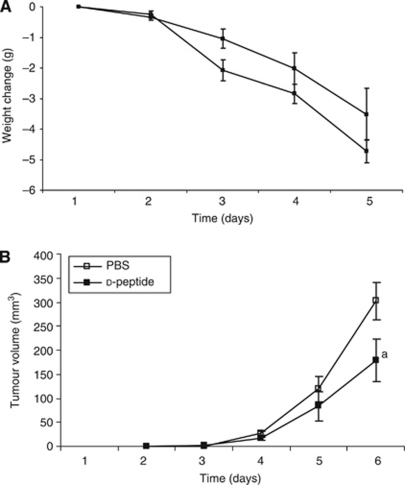
Rate of weight loss (**A**) and tumour growth (**B**) in NMRI mice transplanted with the MAC16 tumour and administered either PBS (100 *μ*l) (□) or D-peptide (100 *μ*g in 100 *μ*l PBS) (▪) daily by i.v. injection. There was no difference in food and water intake between the two groups, and no statistically significant change in the rate of weight loss. Differences from control are shown as a, *P*<0.05.

To determine if this was correct mice bearing the MAC16 tumour were treated daily with either the L- or D-peptide at dose levels up to 100 *μ*g per mouse by i.v. injection. The L-peptide was completely ineffective in the attenuation of the loss of body weight, while the D-peptide-treated animals showed a reduced rate of weight loss (Figure 4A), but this did not reach statistical significance. There was also a tendency to increase lean body mass (D-peptide 22.1±1.5% *vs* PBS 17.4±1.9% *P*=0.09), but again this did not reach statistical significance. There was a small but statistically significant reduction in tumour growth after 6 days treatment with the D-peptide (Figure 4B), but there was no effect with the L-peptide. These results suggest that the peptides may be too hydrophilic to be used as therapeutic agents.

## Discussion

Proteolysis-inducing factor is a sulphated glycoprotein with a short central polypeptide chain, which is derived from dermicidin (Schittek *et al*, 2001), to which are attached sulphated oligosaccharide residues through both *N*- and *O*-glycosidic linkages (Todorov *et al*, 1997). It is strongly catabolic to skeletal muscle by depressing protein synthesis and increasing protein degradation through the ubiquitin-proteasome proteolytic pathway (Eley and Tisdale, 2007). Its presence in urine (Cariuk *et al*, 1997; Williams *et al*, 2004) and tumours (Cabal-Manzano *et al*, 2001) is strongly correlated with the development of cachexia in cancer patients. However, a study in patients with metastatic gastric/oesophageal cancer based on western blotting of urine extracts (Wieland *et al*, 2007) has questioned the role of PIF in weight loss. It is questionable whether these investigators were actually measuring PIF, since the material was relatively abundant, and previous studies (Cariuk *et al*, 1997) had shown PIF to represent just 5 × 10^−4^ percent of the total urinary proteins. The material was later identified as immunoglobulin light chain, which crossreacted with the anti-PIF antibody. This antibody has been shown to be reactive to the carbohydrate chains of PIF (Todorov *et al*, 1997), and may crossreact with other oligosaccharides having a similar antigenic determinant. However, a more recent study (Wang *et al*, 2010) in patients with non-small cell lung cancer using immunohistochemical staining with the same antibody identified PIF in cancer, but not in normal tissue. The positive rate of PIF expression (90%) was significantly higher in patients with weight loss, than those without, and PIF expression was negatively related to survival in medium to advanced stages of disease (II–IV).

To overcome problems with western blotting of PIF, this study reports a new method for the purification of PIF from the MAC16 tumour, which could also be adapted to western blotting of urine extracts. This method is simple and overcomes the problems associated with the purification of a molecule which is only present in tumours at 1 part per 10^7^ of the total tumour protein (Todorov *et al*, 1996a). It utilises the unique characteristics of PIF as a sulphated glycoprotein, which is resistant to pronase and heat denaturation (Todorov *et al*, 1996a). This enables the major tumour proteins to be digested giving a 22 936-fold purification after extraction of PIF by DEAE cellulose (Table 1). It is likely that the oligosaccharide chains in PIF bind around the core peptide protecting it from both heat and proteolytic degradation. A final purification can be achieved by affinity chromatography using either *Triticum vulgaris* (wheat germ agglutinin), which has been shown by lectin blotting studies to show a strong reaction with PIF (Todorov *et al*, 1996b), or anti-PIF monoclonal antibody, previously employed in purification studies (Todorov *et al*, 1996b). The PIF produced by this scheme was biologically active and induced protein degradation in murine myotubes at the same concentration as that previously employed (Whitehouse and Tisdale, 2003).

Previous studies (Todorov *et al*, 2007) have shown that antisera raised against the N-terminal portion of the PIF receptor effectively attenuate protein degradation induced by PIF *in vitro* and protected atrophy of skeletal muscle in mice bearing the cachexia-inducing MAC16 tumour. This occurred through binding of antibody to the receptor, which would sterically exclude PIF from binding. This study reports a similar phenomenon with both the D- and L-forms of the N-terminal 20mer of the PIF receptor, which therefore must be the site to which PIF binds. However, studies in mice bearing the MAC16 tumour show that the peptides were less effective at blocking weight loss than the antibody, possibility due to their hydrophilic nature, which would promote rapid excretion. Despite this the ability of the peptides to block PIF action *in vitro* confirms that the N-terminal region of the receptor is responsible for binding of PIF initiating the signalling pathway leading to protein degradation through the ubiquitin-proteasome proteolytic pathway.

## Figures and Tables

**Table 1:** Purification of PIF from solid MAC16 tumours

**Stage**	**Protein (mg)**	**Recovery (%)**	**Purification (fold)**
Tumour homogenate	2500	—	—
Pronase digest	564	22.56	4.4
DEAE cellulose	2.1	0.08	1190
Heat to 80°C	2.0	0.08	1190
Affinity chromatography (antibody)	0.109	0.004	22 936

Abbreviations: DEAE=diethylamino ethylcellulose; PIF=proteolysis-inducing factor.
